# Adherence to Healthy Lifestyle and the Risk of Function Limitations in Late Life: The Atherosclerosis Risk in Communities Study

**DOI:** 10.3389/fnagi.2021.698699

**Published:** 2021-08-03

**Authors:** Dongze Li, Yu Jia, Jing Yu, Yi Liu, Fanghui Li, Yanmei Liu, Qinqin Wu, Xiaoyang Liao, Zhi Zeng, Rui Zeng, Zhi Wan

**Affiliations:** ^1^Department of Emergency Medicine and National Clinical Research Center for Geriatrics, Research Laboratory of Emergency Medicine, Disaster Medicine Center, West China Hospital, Sichuan University, Chengdu, China; ^2^West China School of Nursing, West China Hospital, Sichuan University, Chengdu, China; ^3^Department of Cardiology, West China Hospital, Sichuan University, Chengdu, China; ^4^Chinese Evidence-Based Medicine Center, West China Hospital, Sichuan University, Chengdu, China; ^5^Health Management Center, West China Hospital, Sichuan University, Chengdu, China; ^6^Department of General Practice and National Clinical Research Center for Geriatrics, West China Hospital, Sichuan University, Chengdu, China

**Keywords:** disability, function limitation, healthy lifestyle, modified risk factor, cohort study

## Abstract

**Background**: Physiological function impairment is the main precursor of assisted living, movement disorder, and disability in the elderly. The relationship between a combination of healthy lifestyle factors and functional limitations is unclear. We investigated the association between healthy lifestyle scores and the risk of functional impairment in community residents.

**Methods**: A total of 10,602 participants (aged 40–64 years) of the Atherosclerosis Risk in Communities (ARIC) study with no history of cardiovascular events and tumors and who came for their fourth visit (1997–1999) were included in the final analysis. Primary outcomes were recorded during the fourth visit; these included impaired lower extremity function, activities of daily living, and instrumental activities of daily living. A logistic regression model was used to test the associations between healthy lifestyle scores and functional impairment. The lifestyle score comprised six factors: healthy diet, moderate alcohol consumption, coffee consumption, physical activity, normal body weight, and no smoking.

**Results**: Among the 10,602 participants with a median follow-up of 9 years, the prevalence rates of impaired lower extremity function, activities of daily living, and instrumental activities of daily living were 50.6%, 14.7%, and 21.6%, respectively. In the adjusted Cox regression model, participants with a healthy lifestyle score of 5 plus 6 had a significant lower risk of impaired lower extremity function (odds ratio = 0.252, 95% confidence interval: 0.184–0.344, *P* < 0.001), activities of daily living (odds ratio = 0.201, 95% confidence interval: 0.106–0.380, *P* < 0.001), and instrumental activities of daily living (odds ratio = 0.274, 95% confidence interval: 0.168–0.449, *P* < 0.001) than did participants with a score of 0. The association of healthy lifestyle scores with impaired activities of daily living and instrumental activities of daily living was stronger for individuals without diabetes than for those with it (*P* for interaction < 0.05). This can be partly explained by the fact that the lowest risk of functional impairment among the participants with diabetes was associated with being overweight.

**Conclusion**: Adherence to an overall healthy lifestyle was associated with a lower risk of physiological function limitation. This study highlights the importance of behavioral interventions in the prevention of disabilities.

**Clinical Trial Registration**: www.ClinicalTrials.gov; Unique identifier: NCT00005131.

## Introduction

With the increasing average life expectancy and the number of elderly people in the United States, it is estimated that by 2030, approximately 20% of the population will be constituted of persons aged ≥65 years, resulting in an increase in the prevalence of chronic physical function impairment (Lubitz et al., 1995). Additionally, it is estimated that between 1985 and 2050, the number of elderly people with functional limitations will triple (Manton, 1997). Physiological impairment is the main precursor of dependent living, movement disorders, and disability in the elderly (Verbrugge and an Jette, 1994). Previous studies have shown that poor physical function is associated with hospitalization, long–term care, and increased mortality (Guralnik et al., 1994; Beswick et al., 2008; Motl and McAuley, 2010). Given that physical function impairment has no specific treatment, it is crucial to control modifiable risk factors to prevent or delay the decline of physical function.

There is increasing evidence of people with healthy lifestyles having a greater likelihood of better physical functions at old age. This healthy lifestyle comprises no smoking, limited alcohol consumption, regular coffee consumption, keeping fit, consumption of a healthy diet, and regular physical activity (Germain et al., 2001; Friedmann et al., 2001; Houston et al., 2005; Maraldi et al., 2009; Hagan et al., 2016; Timmermans et al., 2018; Wang et al., 2021). Furthermore, increasing evidence shows that maintaining multiple healthy behaviors has a greater impact on public health than maintaining a single one (Prochaska et al., 2004, 2008). However, knowledge of the protective effect of multiple healthy behaviors on the risk of functional limitation is limited. In previous studies, a combination of healthy lifestyle behaviors, including no smoking, regular walking, and consumption of plant foods was suggested to considerably increase disability-free survival (Artaud et al., 2013; Zhang et al., 2019). However, there are few studies on the association between a comprehensive healthy lifestyle and functional limitation. It is unknown whether pathways that contribute to disability, such as impaired physical and activity function, are partly inhibited by healthy lifestyles, given that functional limitations are key risk factors for disability (Guralnik et al., 1995; Stuck et al., 1999).

Therefore, we aimed to investigate the association between a healthy lifestyle score and risk of functional impairment in community residents from the Atherosclerosis Risk in Communities (ARIC) study.

## Materials and Methods

### Study Population

This was a secondary analysis of data from the ARIC study. Data were obtained from the public database of the National Heart, Lung, and Blood Institute Biologic Specimen and Data Repository Information Coordinating Center. The ARIC study is a large prospective study covering four communities (Minneapolis, MN; Forsyth County, North Carolina; Washington County, Maryland; and Jackson, MS) in the United States. At baseline (1987–1989), 15,792 community residents aged 45–64 years participated in this study. The participants were followed up every three years. During the follow-up, information on their medical history, lifestyle, cardiovascular events, and other health-related events was collected. The study was approved by the institutional review board at each site. Informed consent was obtained from all the participants. All procedures in this study were done in accordance with the Declaration of Taipei. The study was approved by the institutional review boards at all field centers of the ARIC study and informed consent was obtained from all participants.

**Figure 1:**
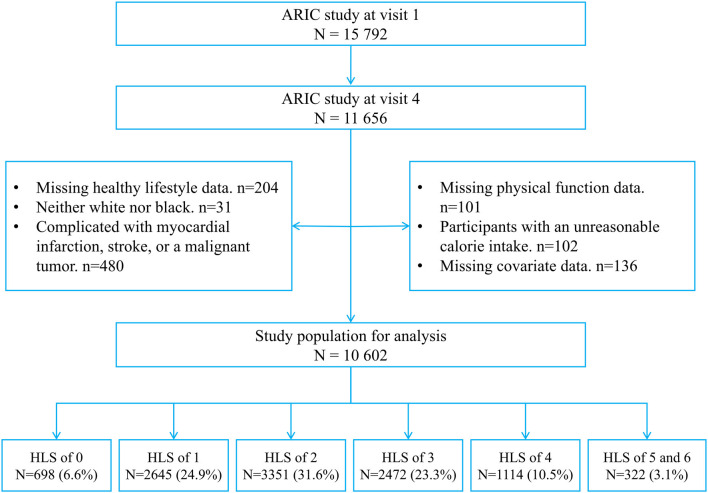
Study flow chart. ARIC, Atherosclerosis Risk in Communities.

A total of 11,656 individuals participated in the fourth follow-up. The following participants were excluded from this study: participants whose healthy lifestyle and physical function could not be evaluated completely (*n* = 305); participants with myocardial infarction, stroke, or a malignant tumor at baseline (*n* = 411; diagnosis of these diseases may lead to changes in lifestyle, and it can significantly contribute to the development of functional limitations and disabilities); participants with an unreasonable calorie intake (≤500 kcal/day or > 5,000 kcal/day, *n* = 102); participants who were neither white nor black (*n* = 31); and participants with incomplete data (*n* = 136). Finally, 10,602 participants were included in this study (Figure 1).

### Definition of the Healthy Lifestyle Score

Six modifiable lifestyle behaviors (healthy diet, moderate alcohol consumption, coffee consumption, physical activity, normal body weight, and no smoking) were components of the healthy lifestyle scores. Their association with functional impairment was also assessed (Germain et al., 2001; Friedmann et al., 2001; Houston et al., 2005; Maraldi et al., 2009; Hagan et al., 2016; Timmermans et al., 2018; Wang et al., 2021).

Dietary intake was assessed using the semi-quantitative food frequency questionnaire by Willett et al. (1987), which included specified portions of 66 foods. The daily nutrient intake was calculated by multiplying the nutrient content of a specific part of each food by its daily intake frequency. Dietary quality was assessed using the Alternative Healthy Eating Index-2010, which consists of 11 components. Of the 11 components, it is encouraged to consume six (vegetables, fruits, whole grains, nuts and legumes, and long chain omega-3 fatty acids), avoid four (sweetened drinks and fruit juice, red and processed meat, trans fats, and sodium), and consume one (alcohol) moderately. A 0–10-point scale is used to score each component; thus, the total Alternative Healthy Eating Index-2010 score ranges from 0 (non-adherence) to 110 (perfect adherence; Wang et al., 2014). It is a high-quality diet, which is useful for the prevention of chronic disease and functional limitations (Chiuve et al., 2012; Hagan et al., 2016). As alcohol consumption was an independent component of the healthy lifestyle score, it was removed from the Alternative Healthy Eating Index-2010. A healthy diet was defined as an Alternative Healthy Eating Index-2010 score ≥40%, as defined in previous studies (van Dam et al., 2008; Wirth et al., 2020).

Moderate alcohol consumption was defined as a daily alcohol intake of 5–15 g for women and 5–30 g for men, according to the guidelines used in the United States (Chiuve et al., 2012).

Coffee consumption was defined as drinking more than two servings of coffee per day. According to previous reports, the general and vulnerable population with a regular intake of at least two cups per day had a lower risk of functional impairment (Machado-Fragua et al., 2019; Wang et al., 2021).

A modified version of the Baecke Questionnaire was used to assess physical activity in the ARIC study (Baecke et al., 1982; Richardson et al., 1995). Participants were asked to report up to four sport activities or exercises they performed most. For each reported sport activity/exercise, additional information on the duration and frequency was recorded. By weighting the product of time and frequency with the task metabolic equivalent corresponding to each activity type, the data were scored again. The final metabolic equivalent of the task was obtained by summing all reported activity types. Based on the guidelines of the World Health Organization, healthy physical activity was defined as a physical activity ≥15 metabolic equivalent of task-hour/week (World Health Organization, 2020).

Height and weight were measured by trained staff and used to calculate the body mass index [BMI; weight (kg) divided by height (m^2^)] during follow-up sessions. A normal body weight was defined as a BMI of 18.5–24.9 kg/m^2^, according to the (World Health Organization, 2004).

In the ARIC study, smoking history was obtained *via* an interviewer-administered questionnaire. Participants were asked whether they were active smokers at the time of the interview or whether they were smokers in the past. We defined never smokers as participants who had never smoked, current smokers as participants who reported active smoking in any questionnaire, and former smokers as participants who had smoked in the past but did not report active smoking at the time of the interview (Nadruz et al., 2016).

Each healthy lifestyle factor was one point; therefore, the range of the healthy lifestyle score was 0–6. Given that lifestyle factors vary with time, the healthy lifestyle score was calculated using data obtained in detail and repeatedly to ensure an accurate representation of the participants’ lifestyles. Information on the Alternative Healthy Eating Index-2010 score, alcohol and coffee consumption, and physical activity were calculated using the average of 3-year repeated measurements. To minimize reverse causality, we used the maximum lifetime BMI.

### Functional Limitations

Physical function was assessed only during the fourth follow-up (1996–1998) using a self-administered questionnaire. The questionnaire comprised 12 activities, and participants were asked to indicate whether they had no difficulties, had difficulties, or had great difficulties doing them or whether they were completely unable to carry out these activities when they were alone, and whether they used aids. As in the third National Health and Nutrition Examination Survey, the 12 activities were divided into lower extremity function, activities of daily living, and instrumental activities of daily living (Ostchega et al., 2000). Lower extremity functions included five activities (Katz et al., 1963; Rosow and Breslau, 1966) walking a quarter-mile; walking up 10 stairs without rest; bending, squatting, or kneeling; lifting or carrying 10 pounds; and standing up from a straight chair without an arm support. Activities of daily living included four activities (Nagi, 1976) walking from one room to another, getting up and out of bed, eating or drinking with glasses, and dressing. Instrumental activities of daily living included three activities (Rosow and Breslau, 1966; Lawton and Brody, 1969) doing housework at home, cooking by oneself, and managing money. Lower extremity function, activities of daily living, and instrumental activities of daily living were classified as “barrier-free” (i.e., participants who had no difficulty in any activity), “impaired” (i.e., participants with any difficulties), or “disabled” (inability to do; Stuck et al., 1999; Houston et al., 2005).

### Covariate Assessment

At baseline, the participants’ socio-demographic information (age, sex, race, income, and education), health behaviors, and prevalence of cardiovascular events and cancer were collected through a standard self-reporting questionnaire. An enzymatic method was used to measure the serum lipid concentration. Hypertension was defined as a systolic blood pressure ≥140 mmHg, diastolic blood pressure ≥90 mmHg, or use of antihypertensive drugs in the last 2 weeks. The sitting arm blood pressure was measured three times after 5 min of quiet rest, with a standardized Hawksley random-zero sphygmomanometer. The final blood pressure value was the average of the second and third readings. Diabetes was defined as a fasting blood glucose concentration ≥126 mg/dl, self-reported history of diabetes, or use of diabetes drugs in the last 2 weeks. The concentration of blood glucose was determined using the improved hexokinase or glucose-6-phosphate dehydrogenase method. Coronary heart disease was diagnosed *via* a self-report questionnaire, medication, and hospitalization history for diseases such as myocardial infarction or coronary revascularization.

### Statistical Analysis

Parametric continuous variables are reported as mean ± standard deviation values; these were compared using ANOVA. Meanwhile, non-parametric continuous variables are reported as medians (25th, 75th percentiles) and were compared using the Mann–Whitney U test. Categorical variables are expressed as frequencies and percentages, and these were compared using the chi-square test.

A logistic regression model was used to assess the relationship of the healthy lifestyle score with impaired lower extremity function, activities of daily living, and instrumental activities of daily living. The group with a healthy lifestyle score of 0 was set as the reference group in the logistic regression model. To further determine whether these relationships were independent of risk factors, the model was adjusted according to age, sex, race, education level (<high school, high school, or >high school), annual household income (<16,000, 16,000–35,000, >35,000 USD), hypertension, diabetes, coronary heart disease, levels of total cholesterol, high-density lipoprotein, low-density lipoprotein, triglycerides, creatinine, and blood glucose, and total calorie intake. A logistic regression analysis was performed after the adjustment for confounding factors to evaluate the association between the healthy lifestyle score and functional limitation in different subgroups of age, sex, race, hypertension, and diabetes; their interactions were tested. Due to the small size of the coronary heart disease subgroups (321, 3%), the model was not used for the subgroups of coronary heart disease.

To explain the different results in the subgroup of diabetes, the correlation of BMI with adjusted odds ratios for functional limitations was analyzed using linear splines with five evenly spaced knots in individuals with and without diabetes.

A two-tailed *P* < 0.05 was considered significant for all tests. All statistical analyses were performed using SPSS version 26.0 (IBM Corp., Armonk, NY, USA) and R software 3.5.0 (Vienna, Austria).

### Data Availability

Data of the Atherosclerosis Risk in Communities study can be obtained from the National Heart, Lung, and Blood Institute Biologic Specimen and Data Repository Information Coordinating Center (BioLINCC)^1^.

## Results

### Baseline Characteristics

Among the 10,602 participants with a median age of 53 years, the participants with a healthy lifestyle score of 0, 1, 2, 3, 4, 5, and 6 were 698 (6.6), 2,645 (24.9), 3,351 (31.6), 2,472 (23.3), 1,114 (10.5), 290 (2.7), and 32 (0.3), respectively. The number of patients with a healthy lifestyle score of 6 was too small for the analysis; therefore, the participants were divided into six groups based on a healthy lifestyle score of 0, 1, 2, 3, 4, and 5 plus 6. Baseline (1987–1989) characteristics are described and compared in Table 1. Participants with a high healthy lifestyle score were more likely to be American Caucasian; have complications like hypertension, diabetes, and coronary heart disease; have a higher level of education and income; have a lower level of cardiovascular biomarkers, and have more healthy behaviors than participants with a low healthy lifestyle score were (*P* < 0.001 for all).

**Table 1:** Baseline (1987–1989) participant characteristics grouped by healthy lifestyle score.

Characteristic	Healthy lifestyle score						
	0	1	2	3	4	5 plus 6	*P*
*N*	698 (6.6)	2,645 (24.9)	3,351 (31.6)	2,472 (23.3)	1,114 (10.5)	322 (3.0)	
**Demographic variables**
Age, years	53.7 ± 5.6	53.9 ± 5.7	53.9 ± 5.7	54.2 ± 5.7	53.9 ± 5.6	52.5 ± 5.7	<0.001
Male sex	362 (51.9)	1,135 (42.9)	1,460 (43.6)	1,107 (44.8)	497 (44.6)	164 (50.9)	<0.001
Race (Black)	311 (44.6)	884 (33.4)	728 (21.7)	269 (10.9)	68 (6.1)	10 (3.1)	<0.001
MET-hour/week	0 (0–5)	0 (0–9)	8.5 (0–20.3)	16.5 (5–30.3)	24.5 (15.8–38)	31.3 (20.3–45.1)	<0.001
Education							<0.001
Less than high school	244 (35.0)	724 (27.4)	590 (17.6)	317 (12.8)	92 (8.3)	14 (4.3)	
High school	201 (28.8)	829 (31.3)	1,099 (32.8)	796 (32.2)	369 (33.1)	74 (23)	
College	253 (36.2)	1,092 (41.3)	1,662 (49.6)	1,359 (55)	653 (58.6)	234 (72.7)	
Smoking							<0.001
Never	1 (0.1)	977 (36.9)	1,578 (47.1)	1,316 (53.2)	644 (57.8)	228 (70.8)	
Ever	444 (63.6)	1,012 (38.3)	1,031 (30.8)	705 (28.5)	289 (25.9)	66 (20.5)	
Current	253 (36.2)	656 (24.8)	742 (22.1)	451 (18.2)	181 (16.2)		
Drinking							<0.001
Never	163 (23.4)	827 (31.3)	903 (26.9)	497 (20.1)	160 (14.4)	16 (5)	
Ever	217 (31.1)	622 (23.5)	573 (17.1)	273 (11)	67 (6)	9 (2.8)	
Current	318 (45.6)	1,196 (45.2)	1,875 (56)	1,702 (68.9)	887 (79.6)	297 (92.2)	
Income, US$							<0.001
	<16,000	201 (28.8)	636 (24)	550 (16.4)	278 (11.2)	96 (8.6)	10 (3.1)
16,000–35,000	294 (42.1)	1,037 (39.2)	1,308 (39)	860 (34.8)	345 (31)	75 (23.3)	
>35,000	203 (29.1)	972 (36.7)	1,493 (44.6)	1,334 (54)	673 (60.4)	237 (73.6)	
**Physiological and lab variables**							
Body mass index, kg/m^2^	30.5 ± 5.1	29.5 ± 5.2	27.8 ± 5.3	26 ± 4.4	24.8 ± 3.7	23.7 ± 2.9	<0.001
Total cholesterol, mmol/l	5.5 ± 1.1	5.6 ± 1.1	5.5 ± 1	5.6 ± 1.1	5.5 ± 1	5.3 ± 0.9	<0.001
HDL-C, mmol/l	1.2 ± 0.4	1.3 ± 0.4	1.3 ± 0.4	1.4 ± 0.4	1.5 ± 0.5	1.5 ± 0.5	<0.001
LDL-C, mmol/l	3.6 ± 1	3.6 ± 1	3.5 ± 1	3.5 ± 1	3.4 ± 0.9	3.3 ± 0.9	<0.001
Triglycerides, mmol/l	1.4 (1–2)	1.3 (1–1.9)	1.3 (0.9–1.8)	1.2 (0.8–1.6)	1.1 (0.8–1.5)	1 (0.7–1.4)	<0.001
Creatinine, mg/dl	1.1 ± 0.2	1.1 ± 0.3	1.1 ± 0.3	1.1 ± 0.2	1.1 ± 0.2	1.1 ± 0.2	<0.200
Blood glucose, mmol/l	6.2 ± 2.2	6.1 ± 2.1	5.9 ± 1.8	5.7 ± 1.5	5.6 ± 1.3	5.5 ± 1.2	<0.001
**Dietary intake**							
Total calories, kcal	1,653 ± 639	1,618 ± 615	1,615 ± 609	1,625 ± 581	1,635 ± 577	1,714 ± 547	<0.071
Coffee intake, servings/day (d)	0.4 (0.1–0.7)	1.1 (0.3–1.8)	1.7 (0.5–2.6)	2.1 (0.9–2.5)	2.5 (1.2–3.1)	2.8 (1.3–3.6)	<0.001
Alcohol intake, g/d	0 (0–1.6)	0 (0–1.5)	0 (0–3.8)	0 (0–9.4)	7.1 (0–12.9)	10.5 (7.5–15.1)	<0.001
Fruits, servings/d	0.9 (0.4–1.7)	1.1 (0.5–1.9)	1.2 (0.6–2)	1.3 (0.6–2.1)	1.5 (0.8–2.2)	1.6 (1–2.1)	<0.001
Vegetables, servings/d	1.3 (0.8–1.9)	1.3 (0.8–2.1)	1.5 (1–2.3)	1.6 (1–2.3)	1.7 (1.1–2.6)	1.9 (1.3–2.6)	<0.001
Red and processed meat, servings/d	1.1 (0.6–1.5)	1 (0.6–1.5)	0.9 (0.5–1.5)	0.9 (0.5–1.4)	0.9 (0.4–1.4)	0.8 (0.4–1.4)	<0.001
Nuts, servings/d	0.5 (0.3–0.7)	0.5 (0.3–0.8)	0.6 (0.3–0.9)	0.6 (0.3–1)	0.6 (0.4–1)	0.6 (0.4–1)	<0.001
Sweetened beverages, servings/d	1 (0.9–1)	1 (0.6–1)	0.9 (0.3–1)	0.6 (0.1–1)	0.5 (0.1–1)	0.6 (0.1–1)	<0.001
Whole grains, g/d	0.4 (0.1–0.9)	0.4 (0.1–0.9)	0.5 (0.1–0.9)	0.6 (0.1–1)	0.6 (0.1–1)	0.6 (0.1–1)	<0.001
Sodium, mg/d	1,549 ± 549	1,535 ± 609	1,514 ± 584	1,462 ± 581	1,441 ± 589	1,434.7 ± 597	<0.001
**Chronic medical conditions**
Hypertension	284 (40.7)	908 (34.3)	860 (25.7)	491 (19.9)	171 (15.4)	30 (9.3)	<0.001
Diabetes mellitus	80 (11.5)	269 (10.2)	242 (7.2)	122 (4.9)	37 (3.3)	10 (3.1)	<0.001
Coronary heart disease	29 (4.2)	115 (4.3)	90 (2.7)		21 (1.9)	5 (1.6)	<0.001

*Values are expressed as N (%), mean ± SD. SBP, systolic blood pressure; DBP, diastolic blood pressure; HDL-C, high density lipoprotein cholesterol; LDL-C, low density lipoprotein cholesterol; MET, metabolic equivalent of task*.

### Healthy Lifestyle Pattern and Adverse Outcome

**Figure 2:**
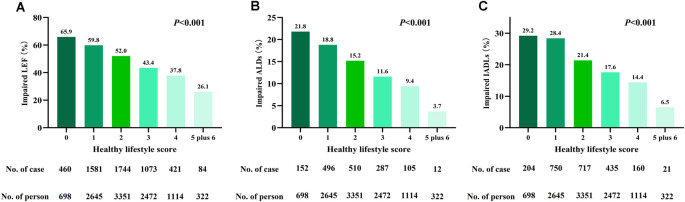
Incidence of impaired **(A)** lower-extremity function (LEF), **(B)** activities of daily living (ADLs), and **(C)** instrumental ADLs (IADLs) grouped by health lifestyle score.

During the median follow-up of 9 years, the prevalence of impaired lower extremity function, activities of daily living, and instrumental activities of daily living was 50.6%, 14.7%, and 21.6%, respectively. Participants with a high healthy lifestyle score had a significantly lower incidence of functional limitations than participants with a low healthy lifestyle score (*P* < 0.001, Figure 2).

In the adjusted logistic regression model (Supplementary Table 1), healthy diet, never smoking, moderate alcohol intake, regular consumption of coffee, physical activity, and normal body weight were all associated with a lower risk of impaired physical function (*P* < 0.05). Among those participants, never smoking, physical activity, and normal body weight were the most significant healthy behaviors for preventing impairment of lower extremity function, activities of daily living, and instrumental activities of daily living (*P* < 0.001). Meanwhile, moderate alcohol intake, regular consumption of coffee, and healthy diet had a protective effect on physical function.

Regarding comprehensive healthy lifestyle scores (Table 2), participants with a healthy lifestyle score of 5 plus 6 had a significantly lower risk of impaired lower extremity function [odds ratio = 0.252, 95% confidence interval (CI): 0.184–0.344, *P* < 0.001], activities of daily living (odds ratio = 0.201, 95% CI: 0.106–0.380, *P* < 0.001), and instrumental activities of daily living (odds ratio = 0.274, 95% CI: 0.168–0.449, *P* < 0.001), than participants with a score of 0. Moreover, participants with a healthy lifestyle score ≥1 had a lower risk of impaired lower extremity function, and participants with a healthy lifestyle score ≥2 had a lower risk of impaired activities of daily living and instrumental activities of daily living than participants with a score of 0 (*P* < 0.001). This means that adherence to only one or two healthy lifestyle factors can decrease the risk of functional limitations. Notably, an increasing healthy lifestyle score was associated with a decreasing odds ratio for impaired function.

**Table 2:** Adjusted odds ratios (OR) for the association of healthy lifestyle score with impaired lower-extremity function (LEF), activities of daily living (ADLs), and instrumental ADLs (IADLs).

HLS	Unadjusted		Model 1		Model 2	
	OR (95%CI)	*P*	OR (95%CI)	*P*	OR (95%CI)	*P*
**Impaired LEF**		<0.001		<0.001		<0.001
0	Ref.	-	Ref.	-	Ref.	-
1	0.769 (0.646–0.916)	<0.003	0.741 (0.618–0.888)	<0.001	0.749 (0.623–0.901)	<0.002
2	0.561 (0.473–0.666)	<0.001	0.571 (0.478–0.683)	<0.001	0.602 (0.502–0.722)	<0.001
3	0.397 (0.333–0.473)	<0.001	0.418 (0.347–0.503)	<0.001	0.451 (0.373–0.545)	<0.001
4	0.314 (0.258–0.383)	<0.001	0.344 (0.279–0.424)	<0.001	0.380 (0.307–0.471)	<0.001
5 plus 6	0.183 (0.136–0.245)	<0.001	0.234 (0.172–0.318)	<0.001	0.252 (0.184–0.344)	<0.001
**Impaired ADLs**		<0.001		<0.001
0	Ref.	-	Ref.	-	Ref.	-
1	0.829 (0.676–1.017)	<0.072	0.840 (0.682–1.034)	<0.100	0.877 (0.708–1.085)	<0.227
2	0.645 (0.526–0.790)	<0.001	0.699 (0.567–0.861)	<0.001	0.765 (0.618–0.949)	<0.015
3	0.472 (0.379–0.587)	<0.001	0.537 (0.428–0.674)	<0.001	0.601 (0.476–0.758)	<0.001
4	0.374 (0.285–0.489)	<0.001	0.447 (0.338–0.591)	<0.001	0.516 (0.388–0.686)	<0.001
5 plus 6	0.139 (0.076–0.254)	<0.001	0.191 (0.104–0.351)	<0.001	0.201 (0.106–0.380)	<0.001
**Impaired IADLs**		<0.001			<0.001	0.008
0	Ref.	-	Ref.	-	Ref.	-
1	0.958 (0.798–1.152)	<0.650	0.990 (0.820–1.197)	<0.921	0.987 (0.814–1.196)	<0.894
2	0.659 (0.549–0.791)	<0.001	0.746 (0.617–0.903)	<0.003	0.780 (0.643–0.946)	<0.012
3	0.517 (0.426–0.627)	<0.001	0.628 (0.512–0.769)	<0.001	0.672 (0.546–0.827)	<0.001
4	0.406 (0.321–0.513)	<0.001	0.525 (0.411–0.671)	<0.001	0.584 (0.455–0.748)	<0.001
5 plus 6	0.169 (0.105–0.271)	<0.001	0.258 (0.16–0.416)	<0.001	0.274 (0.168–0.449)	<0.001

*Model 1: adjusted by age, sex, center-race, education (<high school, high school, or >high school), and annual household income (<16,000, 16,000–35,000, >35,000). Model 2: adjusted by model 1 plus, prevalent hypertension, diabetes, coronary heart disease, total calorie intake, total cholesterol, high density lipoprotein, low density lipoprotein, triglycerides, creatinine, and blood glucose.Unexplained variables are regarded as continuous variables. OR, odds ratio; CI, confidence interval*.

### Subgroup Analysis

**Figure 3:**
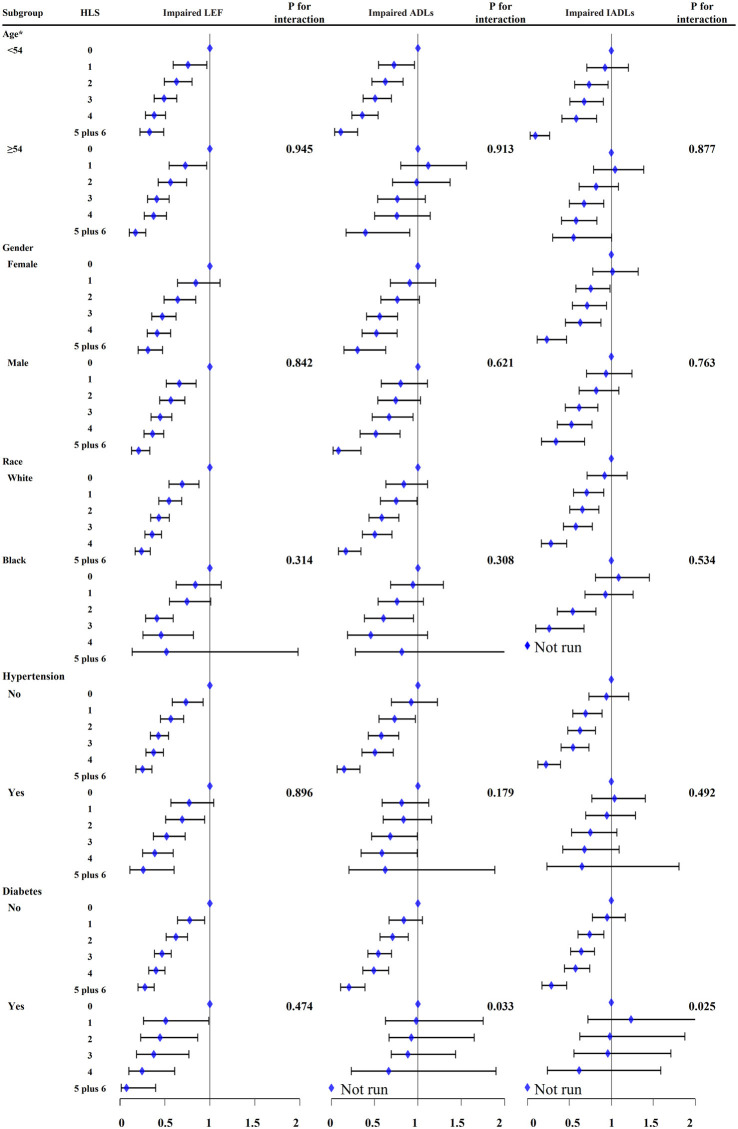
The association between healthy lifestyle score (HLS) with impaired lower-extremity function (LEF), activities of daily living (ADLs), and instrumental ADLs (IADLs) in the subgroup of age, sex, race, hypertension, and diabetes. Some models cannot run because of the very low sample size. Age was divided by median. Multivariable Logistic regression models were adjusted by age, sex, center-race, education (< high school, high school, or >high school), annual household income (<16,000, 16,000–35,000, >35,000), total calorie intake, total cholesterol, high density lipoprotein, low density lipoprotein, triglycerides, creatinine, blood glucose, hypertension, coronary heart disease, and diabetes. CI, confidence interval. OR, odd ratio.

As shown in Figure 3, the association between the healthy lifestyle score and functional limitations was consistent, irrespective of age, sex, race, and hypertension. However, the risk of the healthy lifestyle score being associated with impaired activities of daily living and instrumental activities of daily living was higher for participants without diabetes than for those with diabetes (P for interaction: 0.033 and 0.025, respectively).

### Factor Analysis Between Diabetes and Non-diabetes

In the adjusted logistic regression model, all six binary variables of healthy lifestyle were significantly associated with impaired activities of daily living and instrumental activities of daily living for individuals without diabetes. Meanwhile, the associations of normal body weight with impaired activities of daily living and instrumental activities of daily living were not significant for participants with diabetes. Moreover, only physical activity and never smoked had a significant protective effect on both the risk of impaired activities of daily living and instrumental activities of daily living (Table 3).

**Table 3:** Multivariable logistic regression model for the association of healthy lifestyle factors with impaired activities of daily living (ADLs) and instrumental ADLs (IADLs) for individuals with and without diabetes.

Healthy Lifestyle Factors	Diabetes		Non-diabetes		*P* for interaction
	OR (95%CI)	*P*	OR (95%CI)	*P*	
**Impaired ADLs**					
Healthy diet	0.558 (0.321–0.971)	0.039	0.493 (0.43–0.564)	<0.001	0.945
Moderate alcohol	0.607 (0.342–1.074)	0.087	0.774 (0.66–0.908)	<0.002	0.614
Normal body weight	0.967 (0.701–1.333)	0.836	0.847 (0.753–0.954)	<0.006	0.019
Physical activity	0.637 (0.436–0.931)	0.020	0.743 (0.654–0.845)	<0.001	0.546
Consuming coffee	0.804 (0.565–1.144)	0.226	0.811 (0.721–0.913)	<0.001	0.246
Never smoking	1.346 (0.980–1.850)	0.067	0.627 (0.433–0.91)	<0.014	0.743
**Impaired IADLs**					
Healthy diet	0.796 (0.591–1.072)	0.133	0.818 (0.739–0.905)	<0.001	0.083
Moderate alcohol	0.491 (0.289–0.832)	0.008	0.686 (0.597–0.788)	<0.001	0.545
Normal body weight	0.901 (0.572–1.419)	0.652	0.669 (0.601–0.746)	<0.001	0.002
Physical activity	0.630 (0.454–0.876)	0.006	0.590 (0.530–0.656)	<0.001	0.946
Consuming coffee	0.705 (0.509–0.976)	0.035	0.840 (0.760–0.929)	<0.001	0.841
Never smoking	0.696 (0.489–0.990)	0.044	0.653 (0.582–0.731)	<0.001	0.739

*Multivariable Logistic regression models were adjusted by age, sex, center-race, education (<high school, high school, or >high school), annual household income (<16,000, 16,000–35,000, >35,000), total calorie intake, total cholesterol, high density lipoprotein, low density lipoprotein, triglycerides, creatinine, and blood glucose, hypertension, coronary disease, and diabetes. OR, odds ratio; CI, confidence interval. A healthy diet was defined by the first two quintiles of AHEI-2010. Moderate amount of alcohol was defined by 5–15 g for men and 5–30 g for women. Normal body weight was defined by 18.5 ≤ body mass index < 24.9 kg/m^2^. Physical activity was defined by ≥15 MET-hour/week. Regularly consuming coffee was defined by ≥2 servings/day*.

**Figure 4:**
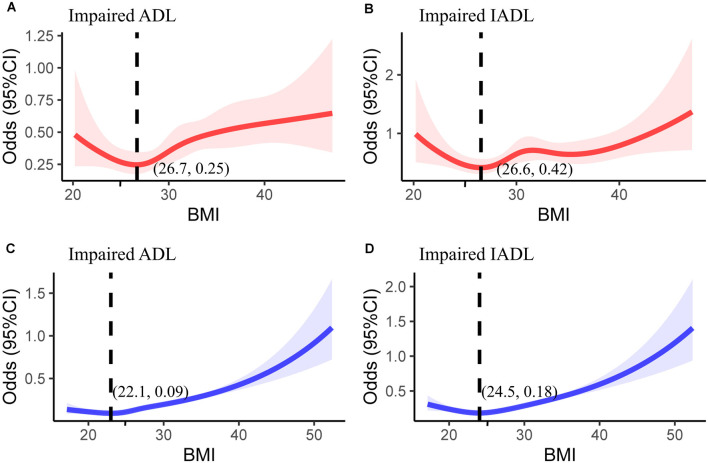
The association between body mass index and odds ratio for impaired **(A)** activities of daily living (ADLs) and **(B)** instrumental ADLs (IADLs) in individuals with diabetes. The same relationship was investigated for impaired **(C)** ADLs and **(D)** IADLs in individuals with diabetes. The adjusted variables of the logistic regression model are consistent with Figure 3. CI, confidence interval.

Given that participants having impaired activities of daily living and instrumental activities of daily living with diabetes had a higher probability of having a normal weight than those without diabetes did (*P* for interaction: 0.019 and 0.002, respectively), linear splines were used to assess the association between BMI and the adjusted odds ratio for impaired functional activities (Figure 4). There were U-shape associations between BMI and the odds ratios for impaired activities of daily living and instrumental activities of daily living in individuals with diabetes. Participants without diabetes who had the lowest risk of impaired functional activities had a normal weight; however, the participants with diabetes who had the lowest risk of impaired functional activity had a BMI of 26 kg/m^2^.

## Discussion

In this community-based cohort study, healthy lifestyle factors including healthy diet (reflected by the first two quintiles of Alternative Healthy Eating Index-2010 diet scores), no smoking, moderate alcohol intake (5–15 g for men; 5–30 g for women), regular consumption of coffee (≥2 cups/day), physical activity (≥15 metabolic equivalent task-hour/week), and normal body weight (BMI: 18.5–24.9 kg/m^2^) were strongly associated with a lower risk of physiological function impairments. Adherence to only one or two healthy lifestyle factors could significantly reduce the risk of functional limitations. The reduction in the risk of functional limitations was more obvious in participants who maintained more healthy lifestyles. Therefore, assuming causal relations, the risk of functional limitations might be reduced by 20–70% in persons with a healthy lifestyle score >2.

In the subgroup analysis, the associations between healthy lifestyle score and impaired functional activities were consistent, irrespective of age, race (Caucasians vs. African Americans), sex, and hypertension; however, these associations were stronger in the participants without diabetes than in those with diabetes. Notably, normal body weight had no significant protective effect on physical function for patients with diabetes. Therefore, we analyzed the linear relationship between the BMI and functional activity limitation. The lowest risk of functional limitations in participants with diabetes is associated with being overweight (World Health Organization standard: BMI of 25–30 kg/m^2^). The lowest risk for participants without diabetes is associated with having a normal weight (18.5–25 kg/m^2^). Most previous studies have found that the extremes of the BMI distribution are associated with a higher risk for disability (Galanos et al., 1994; Ferraro and Booth, 1999; Ferraro et al., 2002) this is consistent with the U-shape associations in this study. Another study reported that overweight was not consistently associated with a higher risk for disability. In addition, one study reported that a BMI between 25 and 35 kg/m^2^ may be protective against functional limitations in men (Friedmann et al., 2001). Therefore, the relationship of BMI with functional impairment and disability is inconsistent. Several mechanisms may explain the relationship between obesity and being underweight. Skeletal stress, protein glycation in connective tissues, or atherogenesis may account for obesity-related disability (Hart and Spector, 1993; Stevens et al., 1993; Pratley et al., 1995). Underweight may be a result of reduced muscle mass and strength, which are important factors of functional impairment Losonczy et al., 1995). Based on a previous study, we minimized reverse causality by using the maximum lifetime BMI for the analysis; however, this may be limited since BMI change patterns had a significant impact on disability development. Although Chiu et al. reported that adults with diabetes having a stable normal weight had the lowest risk of disability, our study suggested that patients with diabetes having a maximum lifetime BMI of 25–30 kg/m^2^ may have a lower risk of functional impairments. This conclusion is consistent with the obesity paradox in previous studies on the relationship between BMI and mortality in patients with diabetes (Costanzo et al., 2015; Zaccardi et al., 2017). Considering that dysfunction and disability are important risk factors for death in patients with diabetes (Lallukka et al., 2016), this may partly explain the obesity paradox in persons with diabetes. Above all, more studies on weight control and physical function are needed, especially for vulnerable populations, such as patients with diabetes.

Many previous studies have reported that moderate alcohol consumption, keeping fit, no smoking, physical activity, regular consumption of coffee, and a high-quality diet are associated with a lower risk of functional limitations and disability (Germain et al., 2001; Friedmann et al., 2001; Houston et al., 2005; Maraldi et al., 2009; Hagan et al., 2016; Timmermans et al., 2018; Wang et al., 2021). However, our results show that individual healthy behaviors (such as coffee intake and moderate alcohol consumption) may only be protective for some physiological functions. Increasing evidence shows that multiple behavioral interventions may have a greater impact on public health than a single behavioral intervention (Prochaska et al., 2008). One research found that a combination of healthy lifestyle behaviors including no smoking, regular walking, and plant food intake may substantially increase disability-free survival (Zhang et al., 2019). Therefore, in line with these previous studies, our study further confirmed that a combination of multiple healthy lifestyle behaviors can strongly prevent a decline in the physical function of the elderly.

Given that health and social care services for elderly persons with disabilities will become a heavy burden and major social concern in the future (Beck and Stuck, 1996) and that functional limitation is a precursor of disability (Verbrugge and an Jette, 1994), changing unhealthy lifestyle behaviors to healthy ones will have massive socio-economic effects on the aging population in future. Therefore, health-related industry associations and social organizations can fully play their role by encouraging the popularization of health sciences *via* media and designing/implementing intervention strategies for different settings in the workplace and community, such as smoking bans and promotion of non-sedentary lifestyles. In addition, the healthy lifestyle score may be used as a simple screening tool for physical function impairment in primary prevention. By identifying high-risk persons, individual lifestyle interventions and risk management can be further implemented by health care professionals. However, the recommendation of a healthy lifestyle for the population is insufficient. Meanwhile, a simple and validated strategy comprising healthy counseling for engaging persons is needed, as it may lead to multiple behavioral changes.

This study had some limitations. First, while detailed and repeated measurements of healthy lifestyle factors were used to calculate the healthy lifestyle score during a study duration of over 9 years, this score relied on self-reported lifestyle factors. Second, only 32 (0.3%) participants had a healthy lifestyle score of 6; thus, the findings for these participants were not conclusive. Third, although associations between the healthy lifestyle score and physical function remained strong and significant after adjustment for several variables, residual confounding (such as body composition, comorbidity, medication, hearing, nutrition, and social support) cannot be totally ruled out in an observational study. Fourth, we excluded people with cardiovascular events and cancer to obtain a relatively healthy cohort, but we cannot be certain that none of the participants in our analysis had a functional impairment at baseline. Fifth, we used the maximum lifetime BMI for analysis and did not consider BMI changes. Therefore, these findings should be further confirmed in other prospective cohorts and randomized-controlled studies.

In general, adherence to an overall healthy lifestyle was associated with a lower risk of impaired lower extremity function, activities of daily living, and instrumental activities of daily living. Given the increasing prevalence of functional limitations and disability, our findings suggest that having at least two healthy lifestyle habits would substantially prevent functional impairments in the elderly and help to reduce the increasing healthcare burdens. Future research is needed to provide more accurate recommendations on healthy lifestyles for elderly individuals, especially for vulnerable populations, such as persons with diabetes.

## Data Availability Statement

The datasets presented in this study can be found in the BioLINCC repository. This data can be found here: https://biolincc.nhlbi.nih.gov/studies/aric.

## Ethics Statement

The studies involving human participants were reviewed and approved by the institutional review boards at all field centers of the ARIC Study and informed consent was obtained from all participants. Written informed consent to participate in this study was provided by the participants’ legal guardian/next of kin.

## Author Contributions

DL, ZZ, and ZW conceived the study design. DL, YJ, FL, YiL, JY, and QW collected the epidemiological and clinical data. DL, YJ, YaL, XL, and ZW summarized data and performed the statistical analysis. DL, YJ, and ZW interpreted the data and drafted the manuscript. ZZ and RZ participated in the design of the study, acquired the data, and helped to revise the manuscript. All the authors contributed to the article, accept responsibility for the entire content of the submitted manuscript and have approved submission.

## Conflict of Interest

The authors declare that the research was conducted in the absence of any commercial or financial relationships that could be construed as a potential conflict of interest.

## Publisher’s Note

All claims expressed in this article are solely those of the authors and do not necessarily represent those of their affiliated organizations, or those of the publisher, the editors and the reviewers. Any product that may be evaluated in this article, or claim that may be made by its manufacturer, is not guaranteed or endorsed by the publisher.

## Footnotes

^1^ https://biolincc.nhlbi.nih.gov
