# Supplementary material for: Adherence to Healthy Lifestyle and the Risk of Function Limitations in Late Life: The Atherosclerosis Risk in Communities Study
Source: Front Aging Neurosci. 2021 Aug 3;13:698699. doi: 10.3389/fnagi.2021.698699 (PMC8369926; doi:10.3389/fnagi.2021.698699)
Supplement: Supplementary file 1 [file Data_Sheet_1.PDF]

Supplementary Table 1. Associations of healthy lifestyle factors with impaired Lower-extremity function (LEF), activities of daily living (ADLs), and instrumental ADLs (IADLs).

Healthy Lifestyle Factors	Unadjusted		Model 1		Model 2	
	OR (95% CI)	P	OR (95% CI)	P	OR (95% CI)	P
**Impaired LEF**						
Healthy diet	0.799 (0.739-0.863)	<0.001	0.817 (0.753-0.886)	<0.001	0.818 (0.753-0.888)	<0.001
Moderate alcohol	0.670 (0.606-0.741)	<0.001	0.898 (0.807-1.000)	0.050	0.921 (0.827-1.026)	0.137
Normal body weight	0.514 (0.473-0.558)	<0.001	0.478 (0.438-0.522)	<0.001	0.518 (0.473-0.567)	<0.001
Physical activity	0.599 (0.553-0.648)	<0.001	0.744 (0.683-0.810)	<0.001	0.756 (0.693-0.823)	<0.001
Consuming coffee	0.821 (0.760-0.887)	<0.001	0.866 (0.819-0.916)	<0.001	0.883 (0.834-0.935)	<0.001
Never smoking	0.679 (0.625-0.738)	<0.001	0.687 (0.631-0.747)	<0.001	0.820 (0.759-0.885)	<0.001
**Impaired ADLs**						
Healthy diet	0.861 (0.799-0.927)	<0.001	0.865 (0.774-0.966)	0.010	0.921 (0.821-1.032)	0.157
Moderate alcohol	0.733 (0.629-0.854)	<0.001	0.913 (0.779-1.069)	0.259	0.954 (0.813-1.120)	0.564
Normal body weight	0.467 (0.410-0.532)	<0.001	0.478 (0.418-0.546)	<0.001	0.516 (0.449-0.593)	<0.001
Physical activity	0.668 (0.595-0.751)	<0.001	0.795 (0.704-0.899)	<0.001	0.809 (0.715-0.916)	0.001
Consuming coffee	0.783 (0.701-0.875)	<0.001	0.890 (0.792-0.999)	0.048	0.948 (0.842-1.066)	0.369
Never smoking	0.529 (0.470-0.596)	<0.001	0.534 (0.474-0.603)	<0.001	0.587 (0.524-0.658)	<0.001
**Impaired IADLs**						
Healthy diet	0.820 (0.746-0.902)	<0.001	0.880 (0.798-0.971)	0.011	0.881 (0.797-0.973)	0.013
Moderate alcohol	0.649 (0.568-0.741)	<0.001	0.836 (0.728-0.961)	0.012	0.865 (0.752-0.996)	0.044
Normal body weight	0.638 (0.575-0.708)	<0.001	0.673 (0.604-0.750)	<0.001	0.746 (0.667-0.834)	<0.001
Physical activity	0.583 (0.527-0.645)	<0.001	0.719 (0.646-0.800)	<0.001	0.731 (0.656-0.814)	<0.001
Consuming coffee	0.801 (0.728-0.881)	<0.001	0.944 (0.854-1.044)	0.263	0.994 (0.897-1.101)	0.905
Never smoking	0.600 (0.542-0.664)	<0.001	0.603 (0.544-0.668)	<0.001	0.673 (0.612-0.741)	<0.001

Model 1: adjusted by age, sex, center-race, education (<high school, high school, or >high school), and annual household income (<16 000, 16 000 to 35 000, >35 000).

Model 2: adjusted by model 1 plus, prevalent of hypertension, diabetes, coronary heart disease, total calorie intake, total cholesterol, high density

lipoprotein, low density lipoprotein, triglycerides, creatinine, and blood glucose.
Unexplained variables are regarded as continuous variables. OR, odds ratio; CI, confidence interval.
All ORs were calculated by comparing healthy lifestyle factors with unhealthy lifestyle factors. Healthy diet was defined by first two quintiles of Alternative Healthy Eating Index-2010. Moderate amount of alcohol was defined by 5-15g for men and 5-30g for women. Normal body weight was defined by 18.5 ≤ body mass index < 24.9 kg/m^2. Physical activity was defined by ≥15 MET-hour/week. Regularly consuming coffee was defined by ≥ 2 servings/day.
